# miR-331-3p and Aurora Kinase inhibitor II co-treatment suppresses prostate cancer tumorigenesis and progression

**DOI:** 10.18632/oncotarget.18664

**Published:** 2017-06-27

**Authors:** Michael R. Epis, Keith M. Giles, Dianne J. Beveridge, Kirsty L. Richardson, Patrick A. Candy, Lisa M. Stuart, Jacqueline Bentel, Ronald J. Cohen, Peter J. Leedman

**Affiliations:** ^1^ Laboratory for Cancer Medicine, Harry Perkins Institute of Medical Research and University of Western Australia Centre for Medical Research, Nedlands, WA, 6009, Australia; ^2^ Department of Anatomical Pathology, Fiona Stanley Hospital, Murdoch, WA, 6150, Australia; ^3^ School of Medicine and Pharmacology, University of Western Australia, Nedlands, WA, 6008, Australia; ^4^ Department of Dermatology, New York University Langone Medical Center, New York, NY, 10016, USA; ^5^ Uropath Pty Ltd, West Leederville, WA, 6007, Australia

**Keywords:** miR-331-3p, prostate cancer, Aurora Kinase inhibitor, co-treatment

## Abstract

RNA-based therapeutics could represent a new avenue of cancer treatment. miRNA 331-3p (miR-331-3p) is implicated in prostate cancer (PCa) as a putative tumor suppressor, but its functional activity and synergy with other anti-tumor agents is largely unknown. We found miR-331-3p expression in PCa tumors was significantly decreased compared to non-malignant matched tissue. Analysis of publicly available PCa gene expression data sets showed miR-331-3p expression negatively correlated with Gleason Score, tumor stage, lymph node involvement and PSA value, and was significantly down regulated in tumor tissue relative to normal prostate tissue. Overexpression of miR-331-3p reduced PCa cell growth, migration and colony formation, as well as xenograft tumor initiation, proliferation and survival of mice. Microarray analysis identified seven novel targets of miR-331-3p in PCa. The 3’-untranslated regions of PLCγ1 and RALA were confirmed as targets of miR-331-3p, with mutation analyses confirming RALA as a direct target. Expression of miR-331-3p or RALA siRNA in PCa cells reduced RALA expression, proliferation, migration and colony formation *in vitro*. RALA expression positively correlated with Gleason grade in two separate studies, as well as in a PCa tissue microarray. Co-treatment using siRALA with an Aurora Kinase inhibitor (AKi-II) decreased colony formation of PCa cells while the combination of AKi-II with miR-331-3p resulted in significant reduction of PCa cell proliferation *in vitro* and PCa xenograft growth *in vivo*. Thus, miR-331-3p directly targets the RALA pathway and the addition of the AKi-II has a synergistic effect on tumor growth inhibition, suggesting a potential role as combination therapy in PCa.

## INTRODUCTION

Prostate cancer (PCa) is the second most frequently diagnosed cancer and cause of cancer related death in men worldwide, accounting for 15% of all male cancers [1, 2]. Recent increases in PCa diagnosis have been in part due to broader awareness of the disease as well as the development of better methods of detection of early stage tumors. Initially, PCas express the androgen receptor (AR) and are dependent on androgens for their growth, providing the basis for androgen ablation therapies. However, the disease inevitably transforms into a hormone refractory or castrate resistant (CR) prostate cancer (CRPC), whereby tumors are resistant to conventional treatments that target the AR pathway and the prognosis is poor. While localized PCa can be treated and potentially cured, later stage CRPC is incurable and treatments or treatment responses are limited and/or short lived [3].

MicroRNAs (miRNAs) are a family of ˜22 nucleotide noncoding RNAs (ncRNAs) that regulate gene expression via specific targeting of the 3’untranslated region (3’UTR) of genes leading to translational repression or message decay [4]. In the previous decade, the involvement of miRNAs in tumor formation and progression of CRPC has become apparent [5]. miRNA dysregulation has been identified in urological diseases, most notably PCa, with more than 40 miRNAs postulated to target diverse oncogenic and tumor suppressor pathways [5, 6], with downstream consequences on a variety of cellular processes that affect tumor growth. Aberrant miRNA regulation in cancer includes overexpression of oncogenic miRNAs (oncomiRs) or loss of expression of tumor suppressor miRNAs. For example, the oncomiR miR-21 is reported to be aberrantly regulated by diverse mechanisms in a variety of cancers [7–14], and elevated serum miR-21 levels in PCa patients are correlated with a CR phenotype [15, 16].

We have previously characterized the capacity of tumor suppressor miRNAs (eg. miR-331-3p; miR-642-5p; miR-7-5p) to regulate the activity of the PI3K/AKT and other pathways in a number of cancer types, including PCa [17–23]. The activity of the PI3K/AKT pathway is controlled upstream by RAS proteins, a family of small GTPases that act as molecular switches for pathways regulating cell proliferation, survival, growth, migration, differentiation, and cytoskeletal dynamism [24]. Additional signaling pathways controlled via activation of RAS proteins include: the RAL (V-Ral Simian Leukemia Viral Oncogene) related proteins RALA and RALB in the RALGEF/RAL pathway (activated via the RALA binding protein 1 (RALBP1)); the protein kinase C (PKC) (activated by way of phospholipase (PLC) proteins eg. PLCγ1); and the ERK (activated through RAF and MEK) pathways [25]. Recent work has focused on inhibiting RAS activated pathways through the use of Aurora Kinase inhibitors (AKi’s), of which there are multiple forms [26]. AKi's have been investigated in a number of clinical trials, including several specifically evaluating the response of prostate tumors [27–30].

The relationship between abnormal miRNA regulation and aberrant RAS expression has been characterised previously in multiple cancers, including PCa [31–35]. Here, we describe two new miR-331-3p targets in PCa, RALA and PLCγ1, which are downstream of RAS in the RALGEF/RAL and PKC activation pathways. Building on our previous studies whereby we identified miR-331-3p as a tumor suppressor miRNA [21, 22], we propose that loss of miR-331-3p expression in PCa is an indicator of advanced disease and has downstream effects on targets within RAS activated pathways, further contributing to disease progression. Further, we demonstrate synergistic PCa growth suppression with a combination of miR-331-3p and an AKi proving a new potential therapeutic avenue in CRPC.

## RESULTS

### miR-331-3p expression is significantly downregulated in prostate tumors

**Figure 1:**
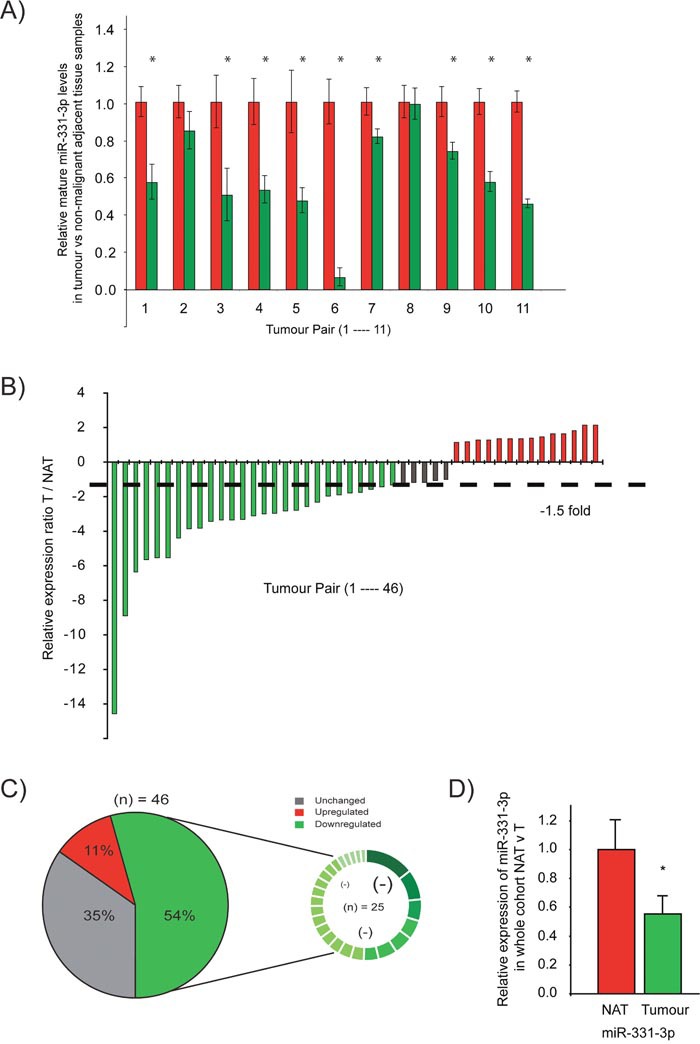
miR-331-3p expression is reduced in PCa tissue relative to a paired non-malignant adjacent tissue (NAT) **(A)** Taqman qRT-PCR detection of miR-331-3p in tumor samples (red bars) relative to a matched non-malignant control sample (green bars) in a pilot cohort study. **(B)** Taqman qRT-PCR detection of miR-331-3p in tumor samples relative to a matched non-malignant control sample in a larger patient cohort study. miR-331-3p expression is colour indicated, where red is significantly increased by > 1.5 fold, green is significantly decreased by > 1.5 fold and grey is unchanged. **(C)** miR-331-3p expression is significantly downregulated in prostate tumors. Pie graph representation of miR-331-3p expression across a large cohort study of 46 patient tumor samples with matched non-malignant control samples. The smaller circle indicates the 25 samples in which miR-331-3p is downregulated, with significant absolute fold changes (<1.5 fold) visually annotated by difference in size and colour gradient within this downregulated subset. **(D)** miR-331-3p expression across all tumor samples compared to non-malignant matched controls in a 46 patients cohort.

Our initial studies focused on evaluation of miR-331-3p expression in PCa compared to adjacent non-malignant prostate tissue. In a pilot cohort of matched nonmalignant prostate vs prostate tumor samples from patients with similar clinical characteristics (age, preoperative PSA, lymph node involvement, Gleason grade), 9 of the 11 samples (81%) exhibited at least a 1.5 fold decrease in miR-331-3p expression in tumor tissue relative to non-malignant prostate (Odds Ratio = 4.5; Figure 1A). From this result, the study was extended to a cohort of 46 patient tumors vs matched nonmalignant prostate tissues, which was the number of patients required to achieve statistical significance (Odds Ratio = 3.00, Power = 0.9). In this cohort, miR-331-3p was down regulated in 33/46 prostate tumor samples when compared to the matched nonmalignant prostate, with 25 of 46 patients (˜55%) showing a 1.5 fold or greater decrease (Figure 1B, 1C). Furthermore, across all specimens, a global down regulation of miR-331-3p within the tumor samples relative to nonmalignant prostate was observed (Figure 1D), and 11 of these cases showed divergent miR-331-3p levels in the tumor and matched non-malignant adjacent tissue (NAT) (Supplementary Figure 1A).

Our findings above were further validated when we interrogated The Cancer Genome Atlas (TCGA) PCa tissue samples for expression of miR-331-3p. We identified an inverse correlation between intratumoral miR-331-3p expression and the clinico-pathologic features of PCa; including Gleason score, tumor stage, ratio of positive to negative lymph nodes and PSA level; suggesting that high tumoral miR-331-3p expression may be beneficial with regards to disease severity (Table 1). To determine whether epigenetic regulation was contributing to the observed miR-3313p down regulation, LNCaP cells were treated with the methylation and acetylation inhibitors TSA (Trichostatin-A) and 5-aza-2’-deoxycytidine (AZA), respectively. The expression of miR-375, a known epigenetically regulated miRNA [67], was increased significantly by methylation and acetylation inhibition, while the expression of miR-331-3p remained unchanged. (Supplementary Figure 2A). Taken together, these data indicate that miR-331-3p is a tumor suppressor in PCa, whereby due to it's frequent downregulation, it is associated with a more aggressive clinical phenotype at the outset.

**Table 1:** Correlation coefficients of intratumoral miR-331-3p expression (prostate cancer) relative to clinicopathologic features from The Cancer Genome Atlas (TCGA)

Variable		High miR-331
**Gleason score**	Cor	-0.125^**^
	n	534
**Tumor stage**	cor	-0.126^**^
	n	534
**Ratio of positive LNs^†^**	cor	-0.151^**^
	n	443
**PSA value**	cor	-0.143^**^
	n	477

^*^Correlation is significant at the p<0.005 level (two-tailed).

^†^ Ratio of positive LNs is the ratio of positive to total lymph nodes examined.

### Effects of miR-331-3p over expression on proliferation, migration, colony formation and xenograft growth

**Figure 2:**
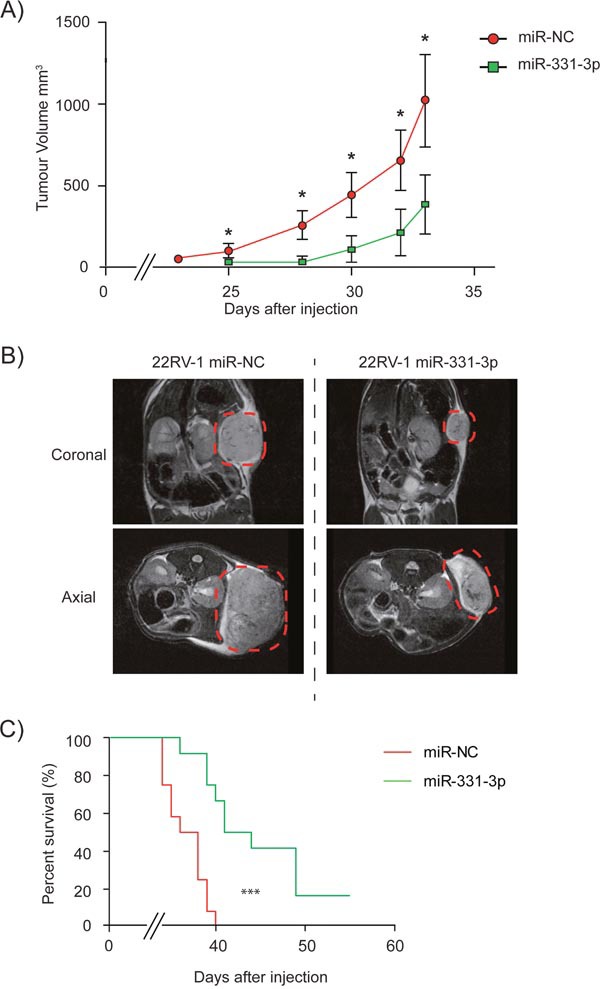
miR-331-3p inhibits PCa xenograft tumor growth Xenografts of 22Rv1 PCa cells transiently overexpressing miR-NC or miR-331-3p on NOD/SCID (NSG) mice. **(A)** Tumors were established by day 25 and size and volume were measured in mice up to day 33. (CI = 0.95; *p < 0.05). **(B)** Coronal and axial MRI images from Day 32 representative mice from both miR-NC and miR-331-3p xenograft groups. Tumor area is highlighted by red dash circle. **(C)** Tumor size end point Kaplan-Meier survival curve of miR-NC vs miR-331-3p xenograft mice. Log-rank (Mantel-Cox) Test ***p<0.0001; Gehan-Breslow-Wilcoxon Test ***p<0.0002.

To evaluate the functional effects of miR-331-3p as a putative tumor suppressor in PCa cells, we initially transiently overexpressed miR-331-3p in DU145 PCa cells and found it reduced proliferation (Supplementary Figure 3A), migration (Supplementary Figure 3B) and colony formation (Supplementary Figure 3C). We next transiently overexpressed miR-331-3p in 22Rv1 cells and transplanted them into NSG mice and noted that miR-331-3p delayed the detection of palpable tumors in NSG mice from day 23 to day 25 compared to miR-NC transfected controls (Figure 2A). In addition, xenograft volume was significantly greater in miR-NC transfected cells at all time points (Figure 2A) and MRI imaging of animals at day 32 post injection indicated marked differences in tumor size (Figure 2B). The end point based on tumor size (1500 mm^3^) was reached first by mice in the miR-NC transfected group at day 33 post injection, and all xenografts formed from these cells reached this end point by day 40 (Figure 2C). In contrast, only 4 of 12 mice injected with miR-331-3p transfected 22Rv1 cells had reached this end point at day 40. When the experiment was extended to 55 days, 10 of the 12 mice in this group had reached the designated tumor end point (Figure 2C). Log-rank (Mantel-Cox) and Gehan-Breslow-Wilcoxon testing revealed the survival of miR-331-3p treated mice was significantly different to miR-NC treated mice (p<0.0001; p<0.0002). These results indicate that transient over expression of miR-331-3p significantly reduced 22Rv1 xenograft growth and that this was associated with increased survival.

### Identification of miR-331-3p target genes

**Figure 3:**
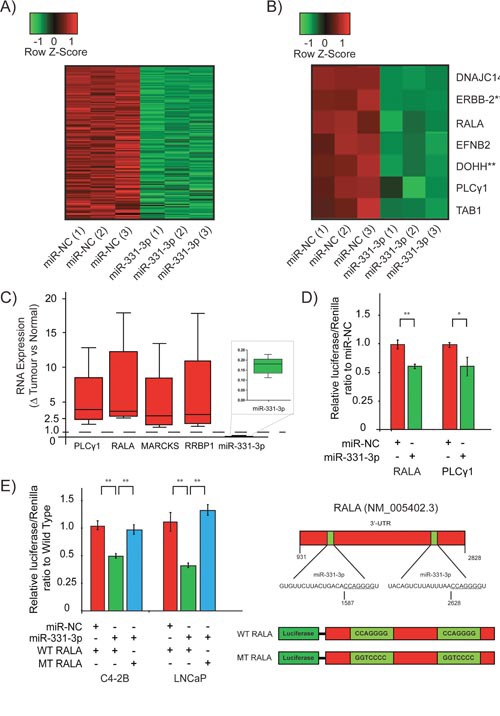
RALA is a direct target of miR-331-3p in PCa cells **(A)** Heat map of all significantly down regulated targets in a microarray study using LNCaP cells transiently expressing miR-331-3p. All targets are represented with or without a predicted miR-331-3p seed region. **(B)** Further stratification of the most down regulated targets identified in the LNCaP/miR-331-3p microarray. ** indicates previously reported targets of miR-331-3p in PCa. **(C)** Box and Whisker plot analyses of PLCγ1, RALA, MARCKS, RRBP1 and miR-331-3p expression in patient tumor v non-malignant prostate tissue. A value of 1.0 is the calculated ratio where the expression of the miRNA or direct target was unchanged between tumor and NAT, and is indicated by the dotted line. **(D)** Luciferase reporter gene analysis of the 3’-UTR of putative miR-331-3p targets PLCγ1 and RALA, in C4-2B PCa cells transiently overexpressing miR-NC or miR-331-3p. **(E)** Luciferase reporter assays of the 3’-UTR of RALA with the two seed regions for miR-331-3p in the 3’-UTR mutated, in LNCaP and C4-2B PCa cells transiently overexpressing miR-NC or miR-331-3p. For all data shown, Error bars = SD; are representative of three independent experiments and *p<0.05, **p<0.005.

To identify the major pathways and genes regulated by miR-331-3p in PCa, we performed microarray analysis of LNCaP cells treated for 24 h with pre-miR-331-3p or pre-miR-NC. We found 148 genes down regulated by at least 1.5 fold in samples treated with pre-miR-331-3p (Figure 3A; Supplementary Table 2). Of these 148 genes, 81 (54.7%) contained miR-331-3p seed regions within their 3’-UTR (p ≤ 3.3 × 10^-8^) based on DAVID and DIANA miR-ExTra analyses. Using Ingenuity Pathway Analysis, we found that each of the 81 genes was over represented in a number of KEGG (Kyoto Encyclopedia of Genes and Genomes) pathways, most notably “Pathways in Cancer”. Seven of the most downregulated genes were studied further (Figure 3B) and the regulation of their expression was independently confirmed by RT-qPCR (Table 2). Included in this cohort were ERBB-2 and DOHH, two targets of miR-331-3p we have described previously [21, 22]. Expression of each of the candidate miR-331-3p targets was evaluated in the 46 PCa cases above, with special attention focused on a subset of samples that indicated the largest change in miR-331-3p expression between a non-malignant adjacent tissue sample and its matched tumor sample. This RT-qPCR analyses indicated that the expression of RALA, PLCγ1, MARCKS and RRBP1 were negatively correlated with that of miR-331-3p in all samples from this subset (Figure 3C). Investigation of PCa cohorts within TCGA revealed miR-331-3p was significantly down regulated in tumor tissue vs normal adjacent benign prostate tissue (Table 3) and also that RALA and PLCγ1 were positively correlated (˜540 samples (cor=0.162, p<0.001); Supplementary Figure 1B). RALA expression was also positively correlated with Gleason score (Table 4). However, RALA and PLCγ1 expression were not significantly correlated with other clinical features of the prostate tumors (Table 4). Additional analysis of data from Taylor *et al* [73] similarly identified that miR-331-3p was significantly down regulated in tumor tissue relative to normal adjacent benign prostate tissue (Table 3) and that RALA expression was positively correlated with Gleason Score (Table 5). To further evaluate this, we immunostained a PCa tissue microarray for RALA expression and found that it progressively increased in tumors of higher TNM stage (Table 6; Supplementary Figure 1C).

**Table 2:** Fold decrease of candidate miR-331-3p target genes from microarray analysis and RT-qPCR detection of the same genes from LNCaP PCa cells overexpressing miR-331-3p

Gene	Array Fold *decrease*	RT-qPCR *decrease*
DNAJC14	2.27	2.98
ERBB-2 [21]	2.15	2.01
RALA	1.94	1.42
EFNB2	1.73	2.38
DOHH [22]	1.57	2.65
PLCγ1	1.56	2.05
TAB1	1.54	1.57

**Table 3:** Average miR-331-3p levels across malignant and adjacent normal prostate tissue in two publicly available cohorts

Cohort	Prostate tissue	n	Average miR-331-3p expression	p-value
Taylor et al. 2010 [73]	Normal	21	9.48	0.007
	Tumor	108	9.23	
Prostate TCGA	Normal	52	17.04	0.009
	Tumor	499	11.71	

P values were calculated using 2-tailed t-testing.

**Table 4:** Correlation coefficients (r) of intratumoral RALA, PLCγ1 expression relative to clinicopathologic features from The Cancer Genome Atlas (TCGA) (prostate cancer)

Variable		High RALA	High PLCG1
**High RALA**	cor		0.161^***^
	n		543
**High PLCG1**	cor	0.161^***^	
	n	543	
**High miR-331**	cor	-0.024	-0.028
	n	543	543
**Gleason score**	cor	0.117^*^	0.040
	n	534	534
**Tumor stage**	cor	0.047	0.016
	n	534	534
**Ratio of positive LNs^†^**	cor	0.014	0.054
	n	443	443
**PSA value**	cor	0.013	0.058
	n	477	477

^*^p <0.005 level, ^***^p <0.0005.

^†^ Ratio of positive LNs: the ratio of positive to total lymph nodes examined.

**Table 5:** Correlation coefficients of intratumoral RALA and PLCγ1 expression (PCa) relative to each other and to Gleason Score or Tumor Stage from Taylor *et al* [73]

Variable		High RALA	High PLCγ1
**High RALA**	r		0.033
	n		218
**High PLCγ1**	r	0.033	
	n	218	
**Gleason score**	r	0.253^***^	0.081
	n	218	218
**Tumor stage**	r	0.119	0.138
	n	200	200

^***^Correlation is significant at the p<0.0005 level (two-tailed).

**Table 6:** RALA expression positively correlates with TNM stage

TNM Stage	RALA expression ratio
T2N0M0	1.25
T2N1M1c	2
T3N0M1	2.25
T4N1M1c	2.375

### RALA and PLCγ1 3’-UTRs are targeted by miR-331-3p

To investigate the interaction between miR-331-3p and RALA and PLCγ1, we evaluated each in luciferase reporter assays. Transient overexpression of miR-331-3p significantly downregulated induced luciferase activity in C4-2B and LNCaP cells, indicating targeting of the RALA and PLCγ1 3’-UTRs by miR-331-3p (Figure 3D; LNCaP data not shown). Mutation of two candidate miR-331-3p seed sites within the 3’-UTR of RALA significantly rescued miR-331-3p induced repressor activity, indicating a direct interaction between miR-331-3p and the RALA 3’-UTR (Figure 3D, 3E). In contrast, mutation of a single conserved miR-331-3p seed site in the 3’-UTR of PLCγ1 did not rescue miR-331-3p effects, suggesting that miR-331-3p activity is either indirect or mediated by alternative seed regions in LNCaP cells (data not shown).

### Targeted degradation of RALA reduces cell proliferation

**Figure 4:**
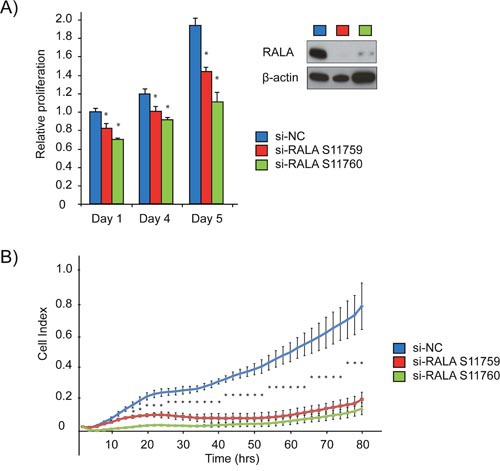
siRNA mediated inhibition of RALA expression reduces proliferation of PCa cells **(A)** LNCaP cells were treated with two different RALA siRNAs and compared to si-NC using cell titre. Immunoblotting for RALA confirmed siRNA mediated depletion. **(B)** Proliferation of LNCaP cells (Cell Index) was measured using the xCELLigence system post RALA siRNA or siNC transfection. Error bars = SD, n=3, *p<0.05.

Focusing on the miR-331-3p target RALA, we assessed the proliferative capacity of PCa cells following siRNA-mediated degradation of RALA. Immunoblotting for RALA indicated at least a 90% reduction in RALA levels in cells transfected with either of two RALA siRNAs (LNCaP cells; Figure 4A). Proliferation was assessed in LNCaP cells +/- RALA siRNA using a CellTiter end point assay or the xCELLigence real time system. RALA siRNA transfected cells exhibited a significant growth reduction as compared to the si-NC transfected cells using both methods of evaluation (Figure 4A and 4B).

### Effects of Aurora Kinase inhibitor II treatment of PCa cells +/- RALA and miR-331-3p

**Figure 5:**
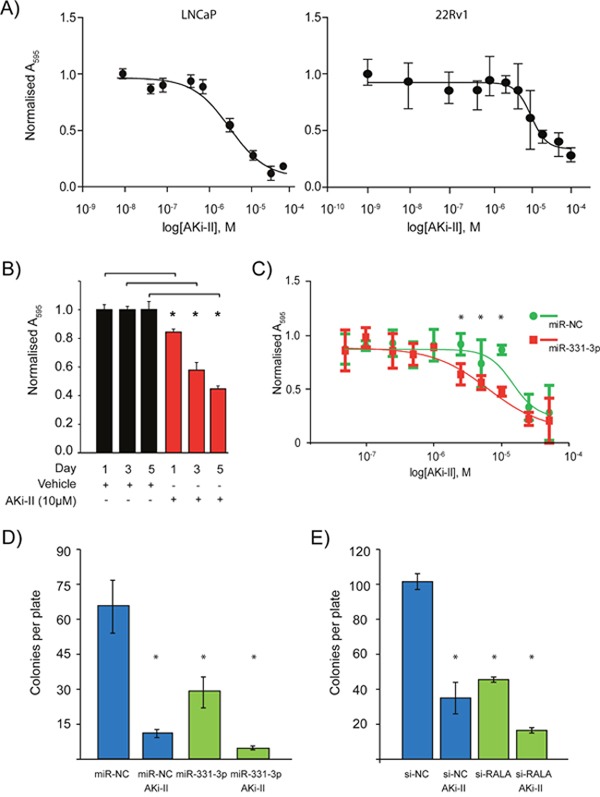
Effects of Aurora kinase inhibitor II treatment of PCa cells +/- RALA and miR-331-3p **(A)** Varying concentrations of AKi-II were used to establish an EC_50_ of the inhibitor in LNCaP and 22Rv1 PCa cells. **(B)** LNCaP PCa cells were treated with 10 μM AKi-II over 5 days and proliferation was measured via cell titre. **(C)** The effect pretreatment of LNCaP PCa cells with miR-331-3p has on the EC_50_ concentration of Aki-II. **(D)** Effects on colony formation of LNCaP PCa cells between miR-NC/miR-331-3p treated cells vs miR-NC/miR-331-3p and AKi-II treated cells. **(E)** Effects of si-RALA treatment on colony formation between si-RALA treated vs si-RALA and AKi-II treated 22Rv1 PCa cells. *p<0.05; CI=0.95; n=3, Error bars = SD.

Given that Aurora Kinase Inhibition could potentially work synergistically with miR-331-3p to inhibit signaling in the Ras-RALA pathway, we next investigated the effects of AKi-II treatment on PCa cells viability. Both LNCaP and 22Rv1 cells were sensitive to the AKi-II with EC_50_ concentrations of 4.57 μM and 10.39 μM, respectively (Figure 5A). Treatment of LNCaP cells with a fixed dose of AKi-II (10 μM) induced a significant decrease in cell proliferation over a 5-day period (Figure 5B). Pretreatment of LNCaP cells with miR-331-3p increased their sensitivity to the AKi-II by ˜57%, suggesting that miR-331-3p targets may be involved in pathways specific to the AKi-II (Figure 5C). Consistent with these results, AKi-II significantly inhibited *in vitro* colony formation and in addition enhanced the inhibitory activity of miR-331-3p or si-RALA treatments on colony formation (Figure 5D and 5E). Using the Bliss Independence Model [74], the combination treatment of cells with miR-331-3p and the AKi-II (Supplementary Table 3) or with si-RALA (Supplementary Table 4) was found to be synergistic in both cases (Figure 5D; Figure 5E ).

### Effects of miR-331-3p and AKi-II pre-treatment of PCa cells on xenograft growth

**Figure 6:**
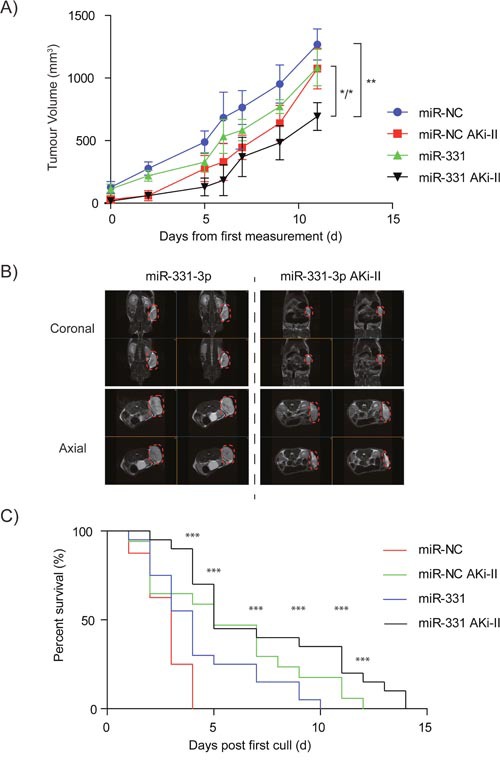
The effects of miR-331-3p and AKi-II pre-treatment of PCa cells on 22Rv1 xenograft growth **(A)** miR-NC and miR-331-3p (+/- AKi-II; 10μM) xenograft NSG mice were monitored over a 33 day period for tumor size and volume. **(B)** Coronal and axial images from Day 33 representative mice from miR-NC and miR-3313p (+/- AKi--II; 10μM) xenograft groups. Tumor area is highlighted by red dash circle. **(C)** End point Kaplan-Meier survival curve of 22Rv1 xenograft mice +/- miR-NC/ miR-331-3p /AKi-II; 10μM). Log-rank (Mantel-Cox) Test; ***p<0.0005. Logrank test for Trend p<0.0011.

To evaluate the effects of combining the AKi-II with miR-331-3p *in vivo*, we established xenografts of miR-331-3p overexpressing 22Rv1 cells that were treated ± Aki-II. As previously observed (Figure 2A), transient overexpression of miR-331-3p in 22Rv1 cells delays the initial detection and subsequent growth of xenografts in comparison to that of control miR-NC transfected cells (Figure 6A; blue vs green). AKi-II pretreatment also reduced xenograft growth, with either AKi-II or miR-331-3p treatments reaching similar tumor volumes at the time of first cull in the miR-NC treated group (Figure 6A). AKi-II pretreatment of miR-331-3p transfected cells further reduced xenograft growth compared to either treatment alone or to that of control miR-NC transfected cells (Figure 6A). MRI revealed that AKi-II treatment of control miR-NC transfected cells significantly reduced the volume of resulting xenografts (not shown), while AKi-II co-treatment further enhanced the inhibitory effects of miR-331-3p transfection on the volumes of the resulting xenografts (Figure 6B). Finally, in miR-NC transfected cell xenografts, tumor volume end points were reached only 4 days after the first cull event, with miR-331-3p transfected cell xenografts taking significantly longer to reach end point at approximately 10 days post first cull (Figure 6C). Two days later (Day 12 post first cull), AKi-II treated (miR-NC transfected) cell xenografts reached end points with the miR-331-3p and AKi-II co-treated group taking the longest to reach end point (extending out to 14 days post first cull) (Figure 6C). Log-rank (Mantel-Cox) testing of all survival curves revealed the survival of AKi-II, miR-331-3p and combination treated mice was significantly different to miR-NC treated mice (p<0.0005), with a Logrank test for trend revealing a significant trend (p<0.0011). Analysing this data using the Bliss Independence Model [74], the combination treatment of the AKi-II with miR-331-3p was found to have an additive effect (Supplementary Table 5). This was opposed to the previous *in-vitro* result using miR-331-3p (Figure 5D) or si-RALA (Figure 5E) in combination with the AKi-II, where we observed a synergistic effect. Overall, these data suggest combining miR-331-3p with an AKi-II will result in increased tumor suppression *in vivo* and supports our previous *in vitro* observations.

## DISCUSSION

These studies demonstrate that miR-331-3p is a tumor suppressor miRNA in PCa and that its loss is associated with a more aggressive disease phenotype. Introduction of miR-331-3p into PCa cells reduces *in vitro* and *in vivo* tumor growth. We identified several new targets for miR-331-3p, one of which, RALA, is a direct target, is downregulated by miR-331-3p in PCa and is an important regulator of PCa growth. We found that an AKi-II was a potent inhibitor of PCa cell growth, and when combined with miR-331-3p further reduced PCa growth *in vivo*.

Over the last decade, global characterisation of miRNAs in cancer, and indeed PCa, has given rise to multiple miRNA signatures associated with a tumorigenic phenotype. miR-331-3p has been associated with chronic and acute lymphoblastic leukemia where it has been described by several separate groups as a potential therapeutic candidate [36–38]. There are a small number of reports that implicate miR-331-3p in the progression or phenotype of PCa [39–41]. Our data contributes to this rapidly growing library of signatures wherein the down regulation or loss of miR-331-3p in PCa appears to contribute to progression and/or phenotype of PCa.

What could be the mechanism for the downregulation of miR-331-3p in PCa? Previously, we investigated whether miR-331-3p in PCa was transcriptionally regulated [21]. We observed that the primary miR-331 transcript was down regulated in a tumor sample compared to a matched non-malignant sample, which resulted in depleted miR-331-3p maturation. This suggested that there was potentially a -pretranscriptional- modification of the primary miR-331 transcript, and ruled out the abnormal maturation of miRNA pathway hypothesis. A common approach to transcript regulation by cells is epigenetic regulation of regions by methylation and acetylation [42–44]. Down regulation of miRNAs, in cancer can be initiated by these epigenetic mechanisms. Here, we sought to investigate whether the observed absence of miR-331-3p in PCa- was due to either DNA methylation or histone deacetylase inhibition and we determined that this was not the case, when compared with a known miRNA, miR-375, whose epigenetic regulation in PCa has been well studied and is transcriptionally repressed via epigenetic mechanisms [67, 45]. Other mechanisms of miRNA down regulation include genomic abnormalities, transcriptional regulation, abnormal maturation pathways and miRNA:miRNA interactions. Pre-transcriptional modifications, such as genomic alterations, are attributed to loss and/or rearrangement of chromosomal locations. The miR-331 gene is situated at chromosomal locus 12q22. Other regions of this chromosome have been found to be lost, mutated or aberrantly expressed in a variety of cancers including pancreatic, gastric, glioblastoma, melanoma and bone marrow/leukemia [46–57]. Therefore, examination of this region for loss or disruption in PCa may provide insight for the down regulation of miR-331-3p expression in our patient sample set.

RALA and PLCγ1 are new targets of miR-331-3p in PCa as determined by bioinformatics, microarray detection, and investigating of a non-malignant vs tumor cohort study. In complimentary reporter gene assays, we validated that RALA is a direct target, evidenced by the wild-type and mutant studies. In contrast, PLCγ1 is an indirect target as the mutation didn't rescue the phenotype in reporter assays. There are two miR-331-3p seed regions within the RALA 3’-UTR compared to one in the PLCγ1 3’-UTR. Both of the RALA 3’-UTR target sequences display complementarity with a seven base pair region (7mer-m8; indicates perfect complementarity between base 2-8 of the seed region [58]) using TargetScan (Release 7.0: August 2015) [59], while the single PLCγ1 miR-331-3p seed region displays a six base pair region, followed by an A (7mer-1A; [58]). Furthermore, context scores of the miR-331-3p sites within the RALA 3’-UTR were higher (context scoring of 80 and 96) compared to the single PLCγ1 site (context score 58). These features combined contribute to the lack of rescue of the mutated PLCγ1 target, and our conclusion that PLCγ1 is an indirect target of miR-331-3p.

Our *in vivo* studies provide robust evidence that replacement of miR-331-3p inhibits PCa growth, consistent with its loss from tumor tissue, and its designation as a tumor suppressor. This effect is potent and long lasting, as cells were transfected only once with the miRNA, prior to being injected intravenously on only one occasion. However, this resulted in a significant biological effect with growth reduction. In fact, we have noticed in similar experiments that transient miRNA expression yields elevated levels of the miRNA in the mouse for up to 17-33 days (unpublished communication). This will be important as the field of RNA-based drug therapy progresses, and variations of miRNAs are developed for delivery as systemic therapy.

The RAL family of small G proteins (RALA and RALB) plays many different roles in tumorigenesis, invasion and metastasis [60–63]. A recent publication indicated that a transiently increased expression of RALA decreased sensitivity to BCNU (Carmustine) and etoposide [60], and increased wound healing [60]. Somewhat contradictory to this, an earlier study by Oxford et al indicated that constitutively active RALA expression inhibited migration [61]. Our observations are consistent with those of Jeon et al, and we propose that the miR-331-3p induced reduction of RALA in our cancer models is cyto “destructive” and anti-migratory. While our model clearly shows that RALA is down regulated with transient miR-331-3p over expression, miR-331-3p also contributes to the down regulation of multiple other transcripts within the cell, as evidenced by our microarray data. Our RALA siRNA data also indicates that in PCa, RALA is pro-tumorigenic, and thus targeting of RALA is likely to be anti-proliferative. Hence, the effects of miR-331-3p on PCa proliferation and migration, are in part, mediated by RALA and/or the RALGEF/Ral activation pathways in PCa cells.

We chose an AKi-II so that we could further inhibit the RAL signaling pathway and determine if we could augment the miR-331-3p-induced reduction of tumor growth. Aurora kinase phosphorylates and activates RALA on a specific site (S194) and is crucial for downstream activation of RALBP1, and promotes tumorigenesis [64]. Thus inhibiting AKII should produce a significant impact on RALA, as it will reduce its functional capacity, and if combined with miR-331-3p it would reduce its tumor level of expression as well. Our studies support this notion, where we were able to get the greatest suppression of tumor growth *in vivo* when miR-331-3p pre-treatment was combined with AKi-II treatment. There are a number of selected publications investigating the efficacy of AKi's specific to PCa [27–30]. Lee et al report that Aurora Kinase inhibitor VX680 reduces cancer cell survival and the effect is enhanced in combination with other chemotherapies (Doxorubicin) [29]. Chieffi et al found that inhibitors of Aurora Kinases, specifically Aurora B, interfere with cell proliferation and may be a therapeutic target for PCa [27]. However, Meulenbeld et al showed that monotherapy of Danusertib, an AKi, showed minimal efficacy in its actions on CRPC, indicating a need for combination therapies [28]. Significantly, a recent finding by Ottman et al reports that the effect of Aurora Kinase inhibitor VX680 (and other current chemotherapeutics) on LNCaP and PC-3 cells is enhanced by the restoration of miRNAs from the miR-17-92a cluster [30]. Herein, we also show that the efficacy of AKi-II treatment may be improved by introduction of miRNAs, as the introduction of miR-331-3p pre-treatment in combination with AKi-II treatment has lasting effects on *in vitro* cell proliferation and colony formation, and most importantly, PCa cell xenograft growth.

Our results lead us to conclude that loss or reduction of miR-331-3p expression in PCa is a common finding and is an indicator of aggressive disease. This down-regulation of miR-331-3p removes suppression of expression on several key downstream targets within the RAS activated and other pathways, which further contribute to disease progression. Specifically, miR-331-3p targets RALA directly reducing its effects on proliferation, migration and tumor growth of PCa cells. Most significantly, when miR-331-3p is combined with existing inhibitors of RAS activated pathways, such as the AKi-II, the combination further reduces *in vivo* PCa tumor growth suggesting a new potential combinatorial approach for treating CRPC.

## Materials and Methods

### Cell culture, plasmid DNA, miRNA precursor, siRNA molecules and inhibitors

LNCaP, DU145 and 22Rv1 cells were obtained from the American Type Culture Collection (ATCC) and cultured at 37°C / 5% CO_2_ in RPMI-1640 supplemented with 10% fetal bovine serum (FBS). C4-2B cells were obtained externally and their phenotype confirmed via STR profiling (Cell Bank Australia, Melbourne). Synthetic miRNA precursor (pre-miR) molecules corresponding to human miR-331-3p (pre-miR-331-3p; Product ID: PM10881), miR-375 (pre-miR-375; Product ID: PM10327) and a negative control miRNA (pre-miR-NC; Negative Control #1, Product ID: AM17110) were sourced from Ambion (Thermo Fisher Scientific). miRNA 3’-UTR target clones for RALA (Product ID HmiT016093), RALA 3’UTR mutant (CS-HmiT016093-MT01-01), PLCγ1 (Product ID HmiT013239) and PLCγ1 3’UTR mutant (CS-HmiT013238-MT01-01) were generated by GeneCopoeia™. Mutation of the candidate miR-331-3p seed sites within the 3’UTR of RALA were nt 1587 CCAGGGG to GGTCCCC and nt 2628 CCAGGGG to GGTCCCC of the complete cDNA sequence; and 3’-UTR of PLCγ1 were nt 612 CAGGGG to GTCCCC (TargetScan Release 7.0: August 2015). Silencer Select siRNAs to RALA (Cat #4392420; Product ID: s11759, s11760), PLCγ1 (Cat #4427037; Product ID: s10631, s10632, s10633) and a negative control siRNA (Cat #4390843; Product ID: Negative Control #1) were sourced from Ambion. Trichostatin A (TSA) (Cat #T-8552) and 5-aza-2’-deoxycytidine (AZA) (Cat #A-3656) were from Sigma and Aurora Kinase inhibitor II (AKi-II) was from Santa Cruz Biotech (Cat #SC-203827).

### RNA extraction, reverse transcription and quantitative polymerase chain reaction (RT-qPCR)

Total RNA was extracted from cell lines and tissue samples using QIAzol reagent as per the manufacturer's instructions (Qiagen). For RNA extractions from tissue, samples were homogenized in QIAzol for 2 × 45 sec pulses using 2.8 mm ceramic beads in a Precellys 24 Homogenizer (Bertin Technologies) prior to RNA extraction. For RT-qPCR, 125 ng RNA was reverse transcribed using a QuantiTect reverse transcription kit (Qiagen). Quantitative PCR was performed in a Corbett 6000 Rotor-Gene thermo cycler (Corbett Research) using QuantiTect SYBR Mix (Qiagen) or Bioline SensiMix (QT605-20) and validated QuantiTect primers (Qiagen) for DNAJC14 (Cat #QT00197043), ERBB-2 (Cat #QT00060746), RALA (Cat #QT00002772), EFNB2 (Cat #QT00024850), DOHH (Cat #QT00235536), PLCγ1 (Cat #QT00048377), TAB1 (Cat #QT00080934), ACTB (Cat #QT01680476), GAPDH (Cat #QT00060746), TUBA1B (Cat #QT00087626) and HPRT1 (Cat #QT00059066). Expression of target mRNAs in all samples were referenced against ACTB, GAPDH, TUBA1B and HPRT1 using GENorm and Normfinder (GenEx software) and relative expression was calculated using the 2^-ΔΔCt^ method. For miRNA expression, 10 ng total RNA was used in TaqMan^®^ miRNA assays (Life Technologies) for hsa-miR-331-3p (Assay ID 000545), hsa-miR-375 (Assay ID 000564) and RNU6B small nuclear RNA (Assay ID 001093) [65] and analyzed in an LC480 Real Time PCR machine (Roche) or a ViiA7 Real Time PCR machine (Life Technologies). The 2^-ΔΔCt^ method was used to determine mature miR-331-3p or miR-375 expression relative to RNU6B small nuclear RNA (snRNA) [66].

### Transfection of siRNA, miRNA precursor molecules and reporter gene assays

PCa cell lines cells were seeded into 6-well or 12-well plates or 10 cm diameter dishes and transfected using Lipofectamine 2000 (Thermo Fisher Scientific). Pre-miRNA molecules were used at final concentrations of 5-50 nM, while siRNA was used at 5-20 nM. Cells were harvested after 24 h for RNA isolation and 3 d for protein extraction. Reporter gene assays were performed as described [21], using bicistronic firefly/*Renilla* luciferase reporter plasmid DNA (RALA 3’-UTR, RALA 3’-UTR mutant, PLCγ1 or PLCγ1 3’-UTR mutant) and 5 nM final concentration of pre-miRNA (pre-miR-331-3p or pre-miR-NC). Lysates were assayed for firefly and *Renilla* luciferase activities using the Dual Luciferase Reporter Assay System (Promega) and a Fluostar OPTIMA microplate reader (BMG Labtech).

### Protein extraction, western blotting and immunohistochemistry

Cytoplasmic protein extracts were prepared and western blotting performed as described [21]. Briefly, protein samples were resolved in NuPAGE 4-12% Bis Tris gels (Thermo Fisher Scientific) and transferred to PVDF membranes (Roche). Membranes were blocked in Tris-buffered saline/Tween 20 (TBST)/5% skim milk and probed with β-actin [AC-15] (Abcam ab6276; 1:10000) and RALA (Cell Signaling Technologies #4799; 1:1500). Detection was performed using horseradish peroxidase-linked anti-mouse IgG (GE Healthcare; Cat #NA931V) or anti-rabbit IgG (GE Healthcare; Cat #NA934V) with Luminata Classico Western HRP substrate (Millipore #WBLUC0100) and ECL-Hyperfilm (VWR #GE HE28-9068-37). Tissue microarray (TMA) slides (Cat. #PR243a) obtained from US Biomax, Inc. (Rockville, USA; Supplementary Table 1). Sections were de-paraffinized in xylene, rehydrated through graded alcohols and subjected to antigen retrieval in citrate buffer pH 6.0 under pressure. Sections were incubated with a RALA antibody (Cell Signaling Technologies #4799; 1:100) for 60 minutes and immunoreactivity visualized using a Dako Envision+ Dual link system-HRP (30 min) and diaminobenzidine (DAKO). Stained slides were independently scored by three researchers.

### DNA methylation and histone deacetylase inhibitors

LNCaP cells were seeded in 6 well plates at 1 × 10^5^ cells/well and treated with vehicle, Trichostatin A (TSA) (200 ng/mL) or 5-aza-2’-deoxycytidine (AZA) (5 μM). RNA was harvested and used for Taqman RT-qPCR detection of miR-331-3p, miR-375 and RNU6B, as described above. miR-375 was used as a positive control miRNA for both TSA and AZA treatment [67].

### PCa cell xenograft model and tumor imaging

22Rv1 cells were transfected as described above with pre-miR-331-3p or pre-miR-NC (50 nM). At 72 hrs post transfection cells were counted and 1 × 10^6^ cells in 150 μL of a 1:1 dilution of RPMI-1640 and Matrigel™-HC (BD BioSciences) was injected subcutaneously into NOD/SCID gamma (NSG) mice (Jackson Laboratory) (12 per group). Where applicable, cells were treated with AKi-II (10 μM) at 48 hr post pre-miR transfection and harvested 24 hrs later for xenograft transplantation [68]. T2 weighted coronal and axial images of NSG mice were generated using a 3.0T MRS 3000 preclinical MRI system at the Australian Cancer Research Foundation Imaging Facility at the Harry Perkins Institute of Medical Research, Perth, Australia.

### Cell proliferation, migration and colony forming assays

Cells transfected with pre-miRNA molecules or siRNAs (as described above) were trypsinized 1-2 days following transfection, plated in 96 well plates at 5000 cells/well and proliferation evaluated using a CellTiter 96 Aqueous One Solution Cell Proliferation System (Promega) in a Fluostar OPTIMA microplate reader (BMG Scientific). The xCELLigence™ system (*In Vitro Technologies*) was used to measure cell proliferation and migration in a real time setting. Pre-miR transfected cells were seeded 1-2 days post transfection into 16 well xCELLigence E-plates for proliferation (5000 cells/well) or 16 well CIM plates for migration (40,000 cells/well), and assayed according to the manufacturer's instructions at 72 h or 24 h, respectively. For colony forming assays, 1000-5000 cells were plated into 10 cm diameter dishes and 3 weeks later colonies were stained with Crystal Violet as previously described [19].

### Prostate tissues

Matched prostate tumor and non-malignant adjacent prostate tissues from 11 patients in a pilot cohort or 46 patients in a subsequent cohort were obtained from Dr. Ronald Cohen (Uropath, Perth, Western Australia). Clinical characteristics of prostate tumor samples were provided and verified to contain >80% tumor by a pathologist.

### cDNA microarray expression profiling and analysis

For microarray analysis, total RNA was isolated from LNCaP cells 24 h following transfection with 30 nM pre-miR-331-3p or pre-miR-NC. The quantity and integrity of extracted RNA was confirmed using a 2100 Bioanalyzer (Agilent Technologies) and gene expression profiling was performed in triplicate by the Australian Genome Research Facility (AGRF; Victoria, Australia) using Human-6 v3 array chips (Illumina). Data normalization was performed by the AGRF. Briefly, raw signal intensity values were subjected to variance stabilization transformation including background correction, log2 transformation and variance stabilization, using the R Bioconductor ‘lumiR’ package (http://www.bioconductor.org/), followed by quantile normalization in Partek^®^ Genomics Suite 6.5 (Partek, Inc).

Ingenuity Pathway Analysis^®^ (Ingenuity System, Inc) and TargetScan (Version 7.1: June 2016) were used to provide metadata on putative miR-331 target genes downregulated by miR-331-3p in the microarray. Specifically, Ingenuity Pathway Analysis^®^ and bioinformatics programs DIANA miR-ExTra (DNA Intelligent Analysis; http://diana.imis.athena-innovation.gr/DianaTools/index.php) [69] and DAVID (The Database for Annotation, Visualization and Integrated Discovery) [70, 71] were used to determine the pathway targets and cellular functions of genes down regulated by miR-331-3p, with corresponding figures produced by PathDesigner^®^. Clustering, volcano and scatter plots showing the distribution of differential gene expression induced by miR-331-3p, were produced from normalized data using the R ‘graphics’ package [72]. A heat map and a Venn diagram were produced with the R ‘gplots’ and the R ‘vennDiagram’ packages, respectively. Microarray expression data has been deposited in the Gene Expression Omnibus under Accession Number GSE96918.

### Publicly available microarray datasets

Cohorts from The Cancer Genome Atlas (TCGA) consisting of varying numbers of prostate cancer patients (see Tables 1, 3, 4 and 5) were analysed by comparing expression of miR-331-3p in tumor tissue vs normal adjacent benign prostate tissue and correlation coefficients of intratumoral RALA, PLCγ1 or miR-331-3p expression (prostate cancer) relative to clinicopathologic features of Gleason score, tumor stage, ratio of positive lymph nodes and PSA values [73].

### Statistical analysis

Analysis of RT-qPCR data was performed using GenEx software (MultiD), at a minimum confidence interval of 95% (CI=0.95) with normality of data confirmed by Kolmogorov-Smirnoff test (KS Test). Gene and miRNA expression in nonmalignant and malignant prostate tissues was compared using the Wilcoxon matched pairs signed rank test. Student's *t*-test was used to analyze luciferase assays, cDNA microarray results, TCGA/public arrays, and PCa xenograft volumes. Synergy or additivity in combined miRNA, siRNA and/or AKi-II experiments was assessed according to the method of Bliss [74], where synergy occurs when E_Bliss_ < E_Observed_. For xenograft survival curves, Log-rank (Mantel-Cox) and Gehan-Breslow-Wilcoxon testing was used for determining significance differences between survival curves and Log-rank test for trends were performed to indicate trend significance of survival curves (Prism 5.0).

## SUPPLEMENTARY MATERIALS FIGURES AND TABLES





## Abbreviations

Abbreviations used are: miRNA: microRNA
PCa: prostate cancer
CRPC: castrate resistant prostate cancer
mRNA: messenger RNA
RT-PCR: reverse transcriptase polymerase chain reaction
RT-qPCR: quantitative RT-PCR
UTR: untranslated region
siRNA: short interfering RNA
pre-miR: precursor miRNA
miR-NC: negative control miRNA
nt: nucleotide
SEM: standard error of the mean
SD: standard deviation
DAVID: (The Database for Annotation, Visualization and Integrated Discovery)
RALA: V-Ral Simian Leukemia Viral Oncogene A
PLCγ1: phospholipase C gamma 1
GAPDH: glyceraldehyde 3-phosphate dehydrogenase
AKi-II: Aurora Kinase Inhibitor II
AKi’s: Aurora Kinase Inhibitors
TSA: Trichostatin A
AZA: 5-aza-2’-deoxycytidine
CI: confidence interval
KS test: KolmogorovSmirnov- test
NAT: Non-malignant adjacent tissue
T: Tumor
TNM: Tumor-Nodes-Metastasis
