# Supplementary material for: miR-331-3p and Aurora Kinase inhibitor II co-treatment suppresses prostate cancer tumorigenesis and progression
Source: Oncotarget. 2017 Jun 27;8(33):55116–34. doi: 10.18632/oncotarget.18664 (PMC5589646; doi:10.18632/oncotarget.18664)
Supplement: Supplementary file 1 [file oncotarget-08-55116-s001.pdf]

miR-331-3p and Aurora Kinase inhibitor II co-treatment suppresses prostate cancer tumorigenesis and progression

SUPPLEMENTARY FIGURES AND TABLES

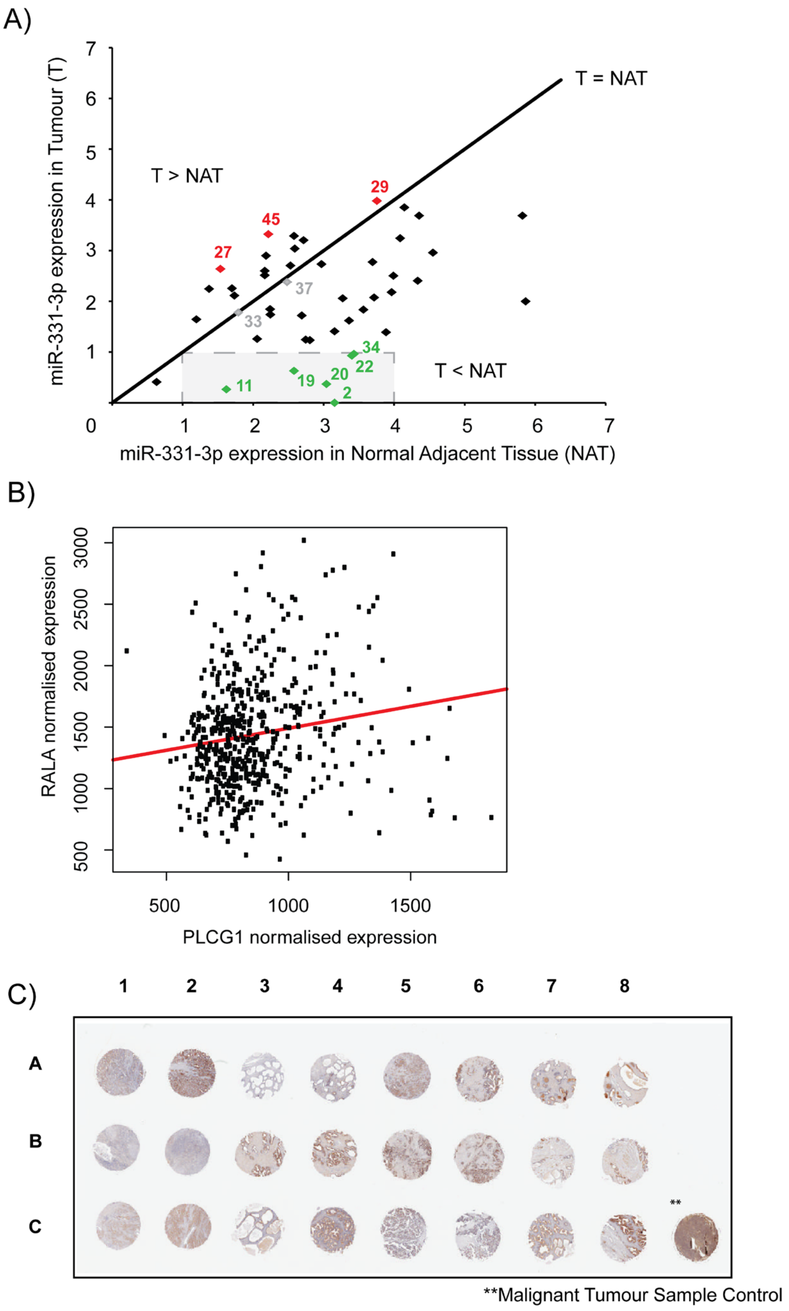

Supplementary Figure 1: (A, B) Scatter plot of the expression ratio of miR-331-3p in Normal Adjacent Tissue (NAT) vs tumour (T) samples. Continuous mid-point line indicates ratio where miR-331-3p expression in NAT is approximately equal to T (ie. T/NAT = 1). Samples below the line are representative of higher miR-331-3p expression in NAT vs matched T tissue. Sample pairs used in analysis at extremities are numbered accordingly and coloured red, grey or green. **(B)** Correlation between RALA and PLCγ1 expression within TCGA PCa cohorts. **(C)** RALA stained tissue microarray slide. Annotations are described in Supplementary Table 1.

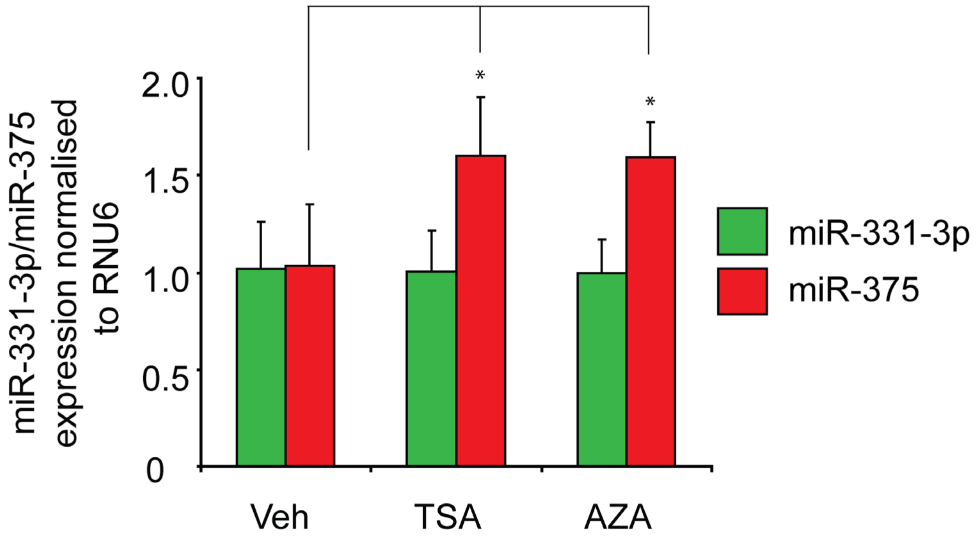

Supplementary Figure 2: (A) LNCaP PCa cells were treated with TSA (Trichostatin A) and 5-aza-2'-deoxycytidine (AZA) and the miR-331-3p and miR-375 transcripts were investigated using Taqman qRT-PCR. Error bars = SD; *p<0.05.

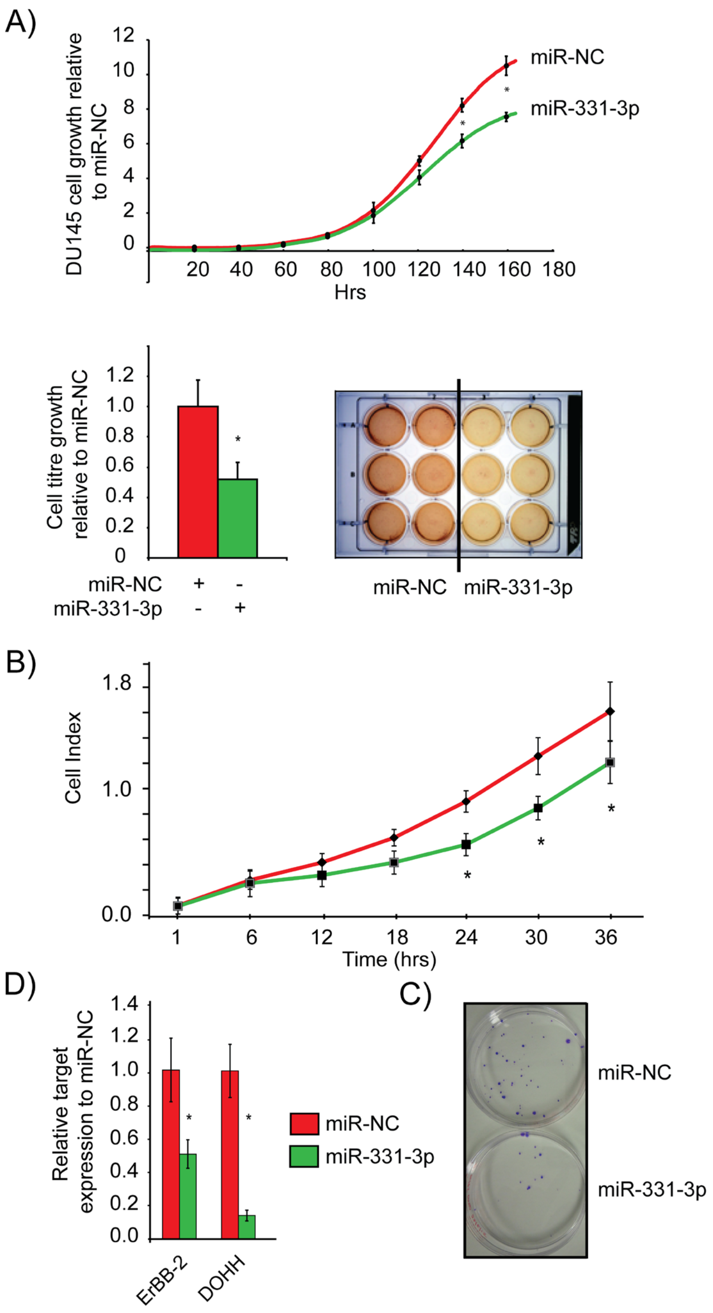

Supplementary Figure 3: miR-331-3p over expression has negative effects on proliferation, migration and colony formation of DU145 PCa cells. **(A)** DU145 cells were transiently transfected with miR-NC or miR-331-3p and their proliferation cell indexes were measured in real time or end point cell titre assays for 160 hours. **(B)** DU145 cells were transiently transfected in with miR-NC or miR-331-3p and their migration cell indexes were measured in real time for 36 hours. **(C)** Colony formation of DU145 miRNC or DU145 miR-331-3p cells visualized 3 weeks post seeding at 1000 cells/plate. **(D)** qPCR analysis of miR-331-3p specific targets confirmed their down regulation (* $p<0.05$).

Supplementary Table 1: Annotation of Tissue Microarray Slide (PR243a; US Biomax)

Pos	No.	Age	Pathology diagnosis	Grade	Gleason Grade	Gleason Score	TNM	Type †
A1	1	69	Adenocarcinoma	3	4	3+4	T2NxM0	Malignant
A2	2	69	Adenocarcinoma	3	4	3+5	T2NxM0	Malignant
A3	3	73	NAT	–	–	–	–	NAT
A4	4	73	NAT	–	–	–	–	NAT
A5	5	60	Adenocarcinoma	1	2	2+1	T4N1M1c	Malignant
A6	6	60	Adenocarcinoma	1	2	2+1	T4N1M1c	Malignant
A7	7	77	NAT	–	–	–	–	NAT
A8	8	77	NAT	–	–	–	–	NAT
B1	9	64	Adenocarcinoma (prostatic tissue)	–	–	–	T2NxM0	Malignant
B2	10	64	Adenocarcinoma	2	2	2+3	T2NxM0	Malignant
B3	11	27	NAT	–	–	–	–	NAT
B4	12	27	NAT	–	–	–	–	NAT
B5	13	64	Adenocarcinoma	2–3	3	3+4	T3NxM1b	Malignant
B6	14	64	Adenocarcinoma	2–3	3	3+4	T3NxM1b	Malignant
B7	15	84	NAT	–	–	–	–	NAT
B8	16	84	NAT	–	–	–	–	NAT
C1	17	75	Adenocarcinoma	1	1	1+2	T2N1M1c	Malignant
C2	18	75	Adenocarcinoma	1	1	1+3	T2N1M1c	Malignant
C3	19	80	NAT	–	–	–	–	NAT
C4	20	80	NAT	–	–	–	–	NAT
C5	21	75	Adenocarcinoma	2	3	3+3	T4N1M1b	Malignant
C6	22	75	Adenocarcinoma	2	3	3+4	T4N1M1b	Malignant
C7	23	21	NAT	–	–	–	–	NAT
C8	24	21	NAT	–	–	–	–	NAT
–	–	58	Malignant Melanoma	–				Malignant

Supplementary Table 2: Down regulated gene list from LNCaP miRNA array (Figure 3A) Genes are ranked from greatest down regulation to least, constrained at -1.5 fold

See Supplementary File 1

Supplementary Table 3: Bliss Independence Model values for calculating synergy/additivity Synergy is concluded if $E_{Observed} < E_{Bliss}$ and Additivity if $E_{Observed} > E_{Bliss}$

Treatment	Colonies (CFU)		Values
miR-NC	66.667		
miR-NC + AKi-II	11	**Ea**	0.165414
miR-331-3p	31	**Eb**	0.466166
miR-331-3p + AKi-II	6.667	$E_{Observed}$	0.097744
		E_{Bliss}	0.554470

Supplementary Table 4: Bliss Independence Model values for calculating synergy/additivity Synergy is concluded if $E_{Observed} < E_{Bliss}$ and Additivity if $E_{Observed} > E_{Bliss}$

Treatment	Colonies (CFU)		Values
si-NC	101.667		
si-NC + AKi-II	35	**Ea**	0.344828
si-RALA S11760	45.333	**Eb**	0.448276
si-RALA S11760+ AKi-II	16.333	$\mathbf{E_{Observed}}$	0.162562
		$\mathbf{E_{Bliss}}$	0.638526

Supplementary Table 5: Bliss Independence Model values for calculating synergy/additivity Synergy is concluded if $E_{Observed} < E_{Bliss}$ and Additivity if $E_{Observed} > E_{Bliss}$

Treatment	Volume (mm^3)	Standard Deviation	Normalised to miR-NC		Values
miR-NC	1268.008	148.0526	1.0		
miR-NC + AKi-II	1085.11	165.0668	0.85576	**Ea**	0.14424
miR-331-3p	1083.779	173.5856	0.85471	**Eb**	0.14529
miR-331-3p + AKi-II	685.7656	112.6894	0.540821	**$E_{Observed}$**	0.459179
				**E_{Bliss}**	0.268574
