# Supplementary material for: miR-331-3p and Aurora Kinase inhibitor II co-treatment suppresses prostate cancer tumorigenesis and progression
Source: Oncotarget. 2017 Jun 27;8(33):55116–34. doi: 10.18632/oncotarget.18664 (PMC5589646; doi:10.18632/oncotarget.18664)
Supplement: Supplementary file 2 [file oncotarget-08-55116-s002.docx]

**Supplementary Table 2.** Down regulated gene list from LNCaP miRNA array (Fig 3A). Genes are ranked from greatest down regulation to least, constrained at -1.5 fold.

| **Gene Symbol** | **p-value (Treated vs. Control)** | **Fold-Change (Treated vs. Control)** |
| --- | --- | --- |
| RRBP1 | 4.40E-07 | -4.02449 |
| VPS25 | 3.35E-06 | -3.12294 |
| LRP3 | 5.62E-05 | -3.02666 |
| FAM152B | 7.97E-07 | -3.0033 |
| HMOX1 | 0.000119497 | -2.81139 |
| C19orf10 | 0.000269323 | -2.74735 |
| BEX2 | 0.000512752 | -2.69653 |
| MT1F | 0.000203865 | -2.63384 |
| TXLNA | 6.71E-05 | -2.49834 |
| IL13RA1 | 0.000616669 | -2.36729 |
| YRDC | 9.50E-05 | -2.337 |
| STX6 | 7.07E-06 | -2.33215 |
| DNAJC14 | 1.35E-06 | -2.27841 |
| WDR55 | 1.06E-05 | -2.23398 |
| ITFG3 | 0.000106577 | -2.23294 |
| ERBB2 | 8.49E-05 | -2.15645 |
| ZMYM6 | 9.49E-05 | -2.14475 |
| NT5C3L | 6.35E-05 | -2.13937 |
| SPRYD5 | 5.34E-06 | -2.13809 |
| STX6 | 0.000190577 | -2.13141 |
| TRIM48 | 0.000673748 | -2.10264 |
| COMMD9 | 0.000951856 | -2.08953 |
| NGFRAP1 | 0.000357638 | -2.08301 |
| BEXL1 | 0.00028762 | -2.04926 |
| OMA1 | 0.00282703 | -2.0401 |
| C12orf52 | 0.00188509 | -1.98726 |
| CCDC56 | 0.000523693 | -1.95495 |
| RALA | 0.000404512 | -1.94324 |
| FAT | 0.000197404 | -1.93234 |
| CXCL10 | 0.00040446 | -1.92461 |
| LOC642362 | 0.000160626 | -1.91529 |
| DDR1 | 0.000208559 | -1.91211 |
| DPP9 | 0.00253838 | -1.90623 |
| CLDND1 | 0.000780582 | -1.90599 |
| KIF5C | 0.000187303 | -1.90245 |
| MARCKS | 3.34E-05 | -1.90149 |
| ANGPT2 | 9.64E-05 | -1.89364 |
| BEX1 | 0.00132958 | -1.88688 |
| DSCR3 | 0.00413294 | -1.87419 |
| DOLPP1 | 0.00200442 | -1.84352 |
| CLDND1 | 0.00208024 | -1.83844 |
| TM4SF1 | 0.000509743 | -1.8284 |
| GUK1 | 0.000196743 | -1.81361 |
| WBP4 | 0.000232736 | -1.79394 |
| PHB2 | 0.00160761 | -1.78935 |
| NGFRAP1 | 0.000102219 | -1.78522 |
| PPM1F | 0.00131718 | -1.77104 |
| ADAMTS1 | 0.000347185 | -1.75328 |
| AKR1C2 | 0.00162714 | -1.74921 |
| HIST1H2BD | 0.000212561 | -1.74785 |
| STAP2 | 0.00204654 | -1.7435 |
| FTL | 0.000348437 | -1.73929 |
| PLD3 | 0.000912085 | -1.73734 |
| EFNB2 | 0.000550685 | -1.73672 |
| UBAP1 | 0.00602179 | -1.73549 |
| STX19 | 0.000217242 | -1.73379 |
| CTDSP2 | 0.00518141 | -1.72931 |
| SYT4 | 0.00107397 | -1.72134 |
| PRCC | 0.00124402 | -1.72024 |
| B4GALT5 | 0.000650451 | -1.71315 |
| HIF1AN | 0.000237971 | -1.70819 |
| COQ2 | 0.00452426 | -1.70753 |
| C2orf25 | 5.56E-05 | -1.70633 |
| UGT2B15 | 0.0618736 | -1.70428 |
| P4HB | 3.21E-05 | -1.696 |
| DIO3 | 0.0147663 | -1.69436 |
| JUP | 0.00167247 | -1.69068 |
| LAMA1 | 0.00850037 | -1.68879 |
| CCL5 | 0.00256638 | -1.68833 |
| BAMBI | 0.00327318 | -1.67909 |
| CCDC127 | 2.06E-05 | -1.67785 |
| KCNN2 | 0.00130124 | -1.66282 |
| VPS4A | 0.00469632 | -1.66258 |
| MGC3196 | 0.00147161 | -1.65759 |
| MT1G | 0.00265816 | -1.65527 |
| IER3IP1 | 0.000565961 | -1.65061 |
| AVPI1 | 0.00124741 | -1.64652 |
| BICD2 | 0.00115299 | -1.64506 |
| SPON2 | 0.000617345 | -1.63437 |
| ST6GALNAC6 | 0.0084814 | -1.63253 |
| CDC42 | 0.000113207 | -1.63029 |
| FTL | 0.000110957 | -1.62488 |
| TOMM22 | 0.000528192 | -1.62381 |
| SERPINI1 | 0.000354254 | -1.6145 |
| IFIT1 | 0.000253283 | -1.61408 |
| TPP1 | 0.00101953 | -1.60975 |
| C7orf44 | 0.000231244 | -1.60823 |
| C2orf25 | 0.00439366 | -1.6031 |
| ENDOGL1 | 0.000593338 | -1.6009 |
| CASP7 | 0.00218623 | -1.59889 |
| SLC44A2 | 0.000131708 | -1.59868 |
| ZNF259 | 0.000612447 | -1.596 |
| ATG16L1 | 0.00626549 | -1.59357 |
| GRWD1 | 0.00900486 | -1.59283 |
| GMFB | 0.000400993 | -1.59247 |
| ATG16L1 | 0.000915676 | -1.59116 |
| RPA2 | 0.0026325 | -1.58982 |
| CCL5 | 0.00141605 | -1.58825 |
| CPSF2 | 0.0193154 | -1.58787 |
| DSC2 | 0.00526429 | -1.5841 |
| TTLL12 | 0.000346568 | -1.5822 |
| MTF1 | 0.00740642 | -1.58114 |
| DOHH | 0.000481903 | -1.57927 |
| IFI27 | 0.00169044 | -1.57711 |
| BEX4 | 0.00333454 | -1.57408 |
| HIBADH | 0.00203064 | -1.57167 |
| FLJ41603 | 0.000419676 | -1.57083 |
| ZNF395 | 0.000940673 | -1.56416 |
| PLCG1 | 0.00550571 | -1.56307 |
| SMARCC1 | 0.000730992 | -1.56302 |
| PARL | 0.00862171 | -1.56226 |
| STAP2 | 0.00045829 | -1.56068 |
| RAP2A | 0.00160353 | -1.55994 |
| MT1A | 0.00329722 | -1.55858 |
| FXR2 | 0.00418575 | -1.55812 |
| PRIC285 | 0.0118633 | -1.55767 |
| CD24 | 0.00560806 | -1.55438 |
| TMEM184B | 0.028211 | -1.55222 |
| MAP3K7IP1 | 0.00233984 | -1.54658 |
| MT2A | 0.00191562 | -1.54275 |
| YRDC | 0.00198895 | -1.54267 |
| COPS7A | 0.00183874 | -1.54264 |
| UGT2B17 | 0.00384763 | -1.53997 |
| AHNAK | 1.64E-05 | -1.53809 |
| PRRG2 | 0.00528543 | -1.5351 |
| LAMP2 | 0.00195613 | -1.53187 |
| IFI6 | 0.0127883 | -1.53074 |
| CMTM7 | 0.00133925 | -1.53072 |
| DSEL | 0.000247406 | -1.52966 |
| GLB1L2 | 0.0110848 | -1.52465 |
| HIST1H2BG | 0.00694183 | -1.52443 |
| GUSBL1 | 0.074526 | -1.52253 |
| LOC728565 | 0.0255022 | -1.52079 |
| SLC30A1 | 0.00543543 | -1.52043 |
| UGT2B7 | 0.00128901 | -1.5201 |
| VIM | 0.00169622 | -1.51968 |
| AGBL5 | 0.000428581 | -1.5155 |
| NIN | 0.000988259 | -1.51539 |
| LOC399939 | 0.0947942 | -1.51517 |
| PCBP4 | 0.00674668 | -1.51414 |
| SMA4 | 0.0307598 | -1.51342 |
| CDC42SE1 | 0.0118304 | -1.5108 |
| SDC1 | 0.00949574 | -1.50961 |
| TMED9 | 0.000128945 | -1.50901 |
| NCDN | 0.0024375 | -1.50791 |
| ZBTB16 | 0.00659742 | -1.50377 |
